# miR-21 expression analysis in budding colon cancer cells by confocal slide scanning microscopy

**DOI:** 10.1007/s10585-018-9945-3

**Published:** 2018-10-25

**Authors:** Kirsten Nguyen Knudsen, Jan Lindebjerg, Alexandra Kalmár, Béla Molnár, Flemming Brandt Sørensen, Torben Frøstrup Hansen, Boye Schnack Nielsen

**Affiliations:** 1 Danish Colorectal Cancer Center South, Vejle Hospital, Part of Lillebaelt Hospital, Beriderbakken 4, 7100 Vejle, Denmark; 2 Department of Clinical Pathology, Vejle Hospital, Part of Lillebaelt Hospital, Beriderbakken 4, 7100 Vejle, Denmark; 3 Institute of Regional Health Research, University of Southern Denmark, Winsløwparken 19,3, 5000 Odense C, Denmark; 4 2nd Department of Internal Medicine, Semmelweis University, Budapest, Hungary; 5 Department of Oncology, Vejle Hospital, Part of Lillebaelt Hospital, Beriderbakken 4, 7100 Vejle, Denmark; 6 Bioneer A/S, Hørsholm, Kogle Allé 2, 2970 Hørsholm, Denmark; 7 Present Address: Department of Pathology, Odense University Hospital, J.B. Winsløws Vej 15, 5000 Odense C, Denmark

**Keywords:** Colon cancer, Confocal slide scanning microscopy, Digital imaging, MicroRNA-21, Multiplex fluorescence, Tumor budding cells

## Abstract

MicroRNA-21 (miR-21) expression in stromal fibroblastic cells in colorectal cancer is well-documented, whereas miR-21 expression in tumor budding cells (TBCs) is poorly described. TBCs are locally invasive carcinoma cells with increased metastatic properties and characteristics of epithelial to mesenchymal transition. This study was conducted to better characterize the expression of miR-21 in TBCs. First, chromogenic miR-21 in situ hybridization (ISH) staining was performed in 58 colon adenocarcinomas with evident TBCs. Then, to obtain unambiguous identification of miR-21 in the TBCs, twenty cases were selected for an additional multiplex fluorescence analysis combining miR-21 ISH with cytokeratin and laminin-5γ2 immunofluorescence. Employing confocal slide scanning microscopy, comprehensive digital images of the invasive front (10–40 mm^2^) were obtained from 16 of the 20 cases, and miR-21 expression was evaluated in cytokeratin-positive TBCs. The high resolution of the confocal digital slide images allowed a detailed examination of the confocal stacks of the multiplex-stained tissue sections. The cases with the highest fraction of miR-21 positive TBCs were all stage III cancers defined by the presence of regional lymph node metastasis. Some of the miR-21 positive TBCs were also laminin-5γ2 positive. The confocal image stacks also revealed that some TBCs were actually directly connected to malignant glands. In conclusion, miR-21 expression was unambiguously identified in TBCs by evaluation of digital slides obtained by confocal slide scanning microscopy. In addition, the digital confocal slides provided a more detailed understanding of local cancer cell invasion by allowing evaluation of the cell structures in three dimensions.

**Electronic supplementary material:**

The online version of this article (10.1007/s10585-018-9945-3) contains supplementary material, which is available to authorized users.

## Introduction

MicroRNAs are short sequences of single-stranded RNA (18–23 nucleotides) that regulate gene expression by binding to mRNA [1]. Some microRNAs act as oncogenic mediators or suppressors of carcinogenesis and cancer cell dissemination [2]. Since its linkage to human cancer in 2005, it is now well-documented that one of the most studied microRNAs, the putative oncogenic microRNA-21 (miR-21), is upregulated in colorectal cancer as well as in most other solid cancers [3–7]. In situ hybridization (ISH) analyses in colorectal cancer tissue have shown that the miR-21 expression is primarily located in the cancer-associated stromal compartment. We as well as others have found that overexpression is related to poor recurrence-free survival in stage II colorectal cancer [8–11]. In these previous studies, we observed that a sub-group of such adenocarcinomas (10–20%) showed focal miR-21 expression in the tumor cells, including in the invasively growing and pro-metastatic tumor budding cells [8, 9].

Tumor budding cells are identified in up to 40% of colorectal cancers as dedifferentiated cells with an invasive appearance [12]. Defined as single cells or groups of less than 5 adenocarcinoma cells, tumor budding cells are located at the tumor periphery (*invasive front*) or intratumorally [13]. They are believed to represent cells in epithelial-to-mesenchymal transition (EMT) because of molecular similarities of this process [12–14]. Several molecular markers have been reported to be characteristic for tumor budding cells, including increased expression of laminin-5γ2, L1CAM and nuclear β-catenin and decreased E-cadherin [14–17]. Tumor budding cells are visible in hematoxylin and eosin (H&E)-stained tissue sections, but are identified more easily with the aid of cytokeratin immunohistochemical labeling [18, 19]. In colorectal cancer, the budding growth pattern is associated with increased lymph node metastases, early recurrence and cancer-related death [20–24]. At the International Budding Consensus Conference in Bern 2016, the assembled histopathologists proposed systematic guidelines for diagnostic use of the tumor budding parameter [25]. This makes the presence of miR-21 positive tumor budding cells of particular interest for future diagnostic applications.

MicroRNA ISH analyses can be performed with high reproducibility and sensitivity in formalin-fixed paraffin-embedded (FFPE) tissue samples [8, 26–28]. This is owing to the microRNAs being well conserved in FFPE samples and retrievable from the protein complexes [29, 30]. In our experience, conventional chromogenic ISH (CISH) may provide only limited histological information in the attempt to study miR-21 positive tumor budding cells. To identify single or small groups of miR-21 positive tumor budding cells is particularly challenging due to the multiple miR-21 positive fibroblastic stromal cells at the invasive front. Thus, the current study was conducted to provide a method for better characterization of the miR-21 expressing tumor budding cells using combined miR-21 ISH and immunofluorescence staining of cytokeratin and laminin-5γ2 and evaluation of the expression patterns on digital slides.

The use of conventional epifluorescence microscopy to evaluate multiplex fluorescence stained slides can be a time-consuming process. It involves continuous changing of lenses and filter sets as well as image acquisition with exposure time optimization for all fluorophores. In addition, formalin fixation may cause relatively high unspecific autofluorescence and high background fluorescence noise [31]. Furthermore, the fluorescence signals fade over time, which prevents more exhaustive examination and re-inspection of the slides after some time. Confocal slide scanning microscopy (CSSM) enables the generation of multifluorescence digital slides based on the so-called *extended focus principle* that comprises the highest intensity single pixels of individual fluorescence signals from the serial confocal image stacks. By introducing structured illumination for the confocal imaging [32, 33] discrete output (20–25 nms) solid state light sources, narrow bandwidth filter sets, and digital gain of in-focus fluorescence signals, it is possible to detect small size, low emission fluorescence signal by reducing the ratio of autofluorescence and minimizing fluorescence bleed through. In epifluorescence microscopy the autofluorescence signal of the FFPE tissue section is emerging from the whole thickness of the section. In addition, the acquired digital slides can be examined using software-assisted digital zoom and focus with the option to evaluate single or more fluorescence channels at the same time. The evaluation of single focal planes also allows visualization of structural details in the tissue that are otherwise undetectable in images obtained using conventional optics.

In the present study, we obtained confocal digital slides comprising four fluorophore stains covering the invasive front in selected colon adenocarcinomas in order to characterize and quantify the presence of miR-21 positive tumor budding cells.

## Materials and methods

### Tissue specimens

The study material consisted of 58 FFPE stage II (n = 36) and III (n = 22) colon cancers diagnosed in the period from 2000 to 2008 at the Department of Clinical Pathology, Vejle Hospital, Denmark. Details of the selection process of the cohort have previously been published elsewhere [34]. In brief, only conventional pT3 adenocarcinomas with at least 10 buds, each containing a maximum of four tumor cells were included. The tumor budding evaluation was performed on pan-cytokeratin stained slides with a 20 × objective, and all cases were then allocated into high and low budding groups based on the approach first described by Karamitopoulou et al. [35]. Information on subsequent development of distant, malignant dissemination was retrieved via medical charts. Clinico-pathologic characteristics are shown in Table S1 and have previously been published elsewhere [34]. A subset of 20 specimens was selected for multiplex fluorescence analysis as described previously [34]. The selection comprised cases with without subsequent development of distant metastases and included cases with high budding (n = 13) and low budding (n = 7). Sixteen of these cases were evaluable by the multiplex fluorescence technique. Three cases were excluded because of tissue folds or detachment from the slides and one case showed extensive necrosis (Table S1). The study was registered at the Danish Data Protection Agency and was approved by the Regional Committees on Health Research Ethics (ID# S-20120075). The Danish Registry of Human Tissue Utilization was consulted before utilization of any tissue samples.

### Chromogenic in situ hybridization and scoring evaluation

The CISH assay was performed on 5 µm thick sections with 30 nM double-FAM-labeled miR-21 (TCAACATCAGTCTGATAAGCTA, RNA Tm, 83 °C; 32% Locked Nucleid Acid (LNA), Exiqon, Vedbæk, Denmark) as described previously [8, 27]. The specificity of the miR-21 ISH signal has been analyzed in detail previously with inclusion of both negative and positive control probes (7). The slides were evaluated and the overall staining was scored semi-quantitatively according to miR-21 staining intensity (0 = negative, 1 = weak, 2 = strong), and proportion of stained cells (0 = < 10%, 1 = 10–50% and 2 > 50%). The total score was determined by adding the two scores, and the total score was then divided into two categories: low miR-21 expression (sum ≤ 2) and high expression (sum > 2). The evaluation was performed individually for the stromal cells in the tumor center and the periphery and the cohesive adenocarcinoma cells in the center and periphery, while the evaluation for tumor budding cells was only performed for those at the invasive front. The clinical data was not blinded during the evaluation.

### Multiplex fluorescence staining

Five µm thick FFPE sections were subjected to a combined ISH and IHC fluorescence staining procedure as described elsewhere in detail [36]. In brief, air-dried, deparaffinized sections were treated with 25 µg/ml proteinase-K for 10 min at 37 °C. Hybridization was performed with 20 nM double-FAM-labeled LNA probe for miR-21 (TCAACATCAGTCTGATAAGCTA; RNA Tm, 83 °C; 32% Locked Nucleid Acid) in Exiqon hybridization buffer (Exiqon, Vedbæk, Denmark) at 55 °C for 1 h, followed by probe detection with peroxidase-conjugated anti-FAM (Roche, Basel, Switzerland). The sections were incubated in TSA-Cy5 substrate (Perkin Elmer, Waltham, MA, USA) for 10 min at room temperature, washed in PBS and incubated for 10 min with 3% hydrogen peroxide. The ISH process was then followed by two consecutive immunofluorescence procedures. First, the sections were incubated with mouse-anti-cytokeratin, clones AE1/AE3 (diluted 1:200, Dako, Glostrup, Denmark) overnight at 4 °C, detected with HRP-conjugated anti-mouse (Jackson ImmunoResearch, West Grove, PA, USA) and incubated in TSA-FITC substrate (Perkin Elmer, Waltham, MA, USA) for 7 min at room temperature. After brief washes in PBS, the sections were treated with glycin/SDS-buffer [37] to elute all antibodies. Secondly, the sections were incubated with mouse-anti-laminin-5γ2-chain, clone D4B5 (diluted 1:200, Merck Millipore, Billerica, MA, USA) at room temperature and detected with Cy3-conjugated anti-mouse (Jackson ImmunoResearch, West Grove, PA, USA). Finally, the sections were mounted with DAPI-containing mounting medium, ProLong Gold (Thermo Fisher Scientific, Waltham, MA, USA).

### Confocal scanning microscopy

Initially, one or two areas of interest (6 and 14 cases, respectively) comprising the invasive front with high degree of tumor budding was delineated on the H&E stained slides by two senior pathologists (JL and FBS). Confocal slide scanning was then performed on the multiplex fluorescence-stained slides using a Pannoramic confocal scanner (3DHISTECH Ltd., Budapest, Hungary). The system was equipped with a Lumencor Spectra X solid-state discrete output light engine (Lumencor, Beaverton, OR). The following LEDs were applied in the excitations: DAPI 390/22 nms, 520 mW, Cy3 555/28 nms, 370 mW, Cy5 635/22 nms, 510 mW, FITC 475/28, 530 mW. The confocal imaging is made by the laser-free structured illumination unit (Aurox, Abingdon, UK). First, the delineated areas identified on the H&E-stained slides were applied on standard fluorescence pre-scanned digital whole slides obtained from the DAPI fluorescence signal using a 20 × lens with 0.8 numeric aperture (NA, Zeiss, Oberkochen, Germany). Confocal slide scanning was then performed using a 40 × water immersion objective [C-Apochromat (W), Zeiss, Oberkochen, Germany] with 1.2 NA providing 220 nm FWHM optical XY resolution at 500 nm wavelength. The confocal slide scanner was equipped with a Confocal PCO edge 5.5 camera, and a 1 × camera adapter magnification. For slide scanning, single pass filters were used as follows: DAPI (exciter: 387 nm/11 nm, emitter: 440 nm/40 nm, dichroic: 410 nm), FITC (exciter: 485 nm/20 nm, emitter: 521 nm/21 nm, dichroic: 504 nm), TRITC/Cy3 (exciter: 559.5 nm/25 nm, emitter: 607 nm/34 nm, dichroic: 582 nm), Cy5 (exciter: 649.5 nm/13 nm, emitter: 700 nm/45 nm, dichroic: 669 nm) filter sets (Semrock, New York, USA). We used 100–300 ms exposure time and applied digital gain on the camera side (varying from 0 to 3) for the faster imaging and additional confocal gain (varying from 1.0 to 2.0) depending on the intensity of the four individual fluorescence signals. The confocal gain is a multiplying factor of the pixels in the confocal plane after deduction by the non-confocal plane image pixels, which helps to increase the contrast of the confocal image components. The approximately 5 µm thick sections were scanned at confocal layers of 0.4 µm distance resulting in stacks of up to 12 confocal layers.

**Fig. 1:**
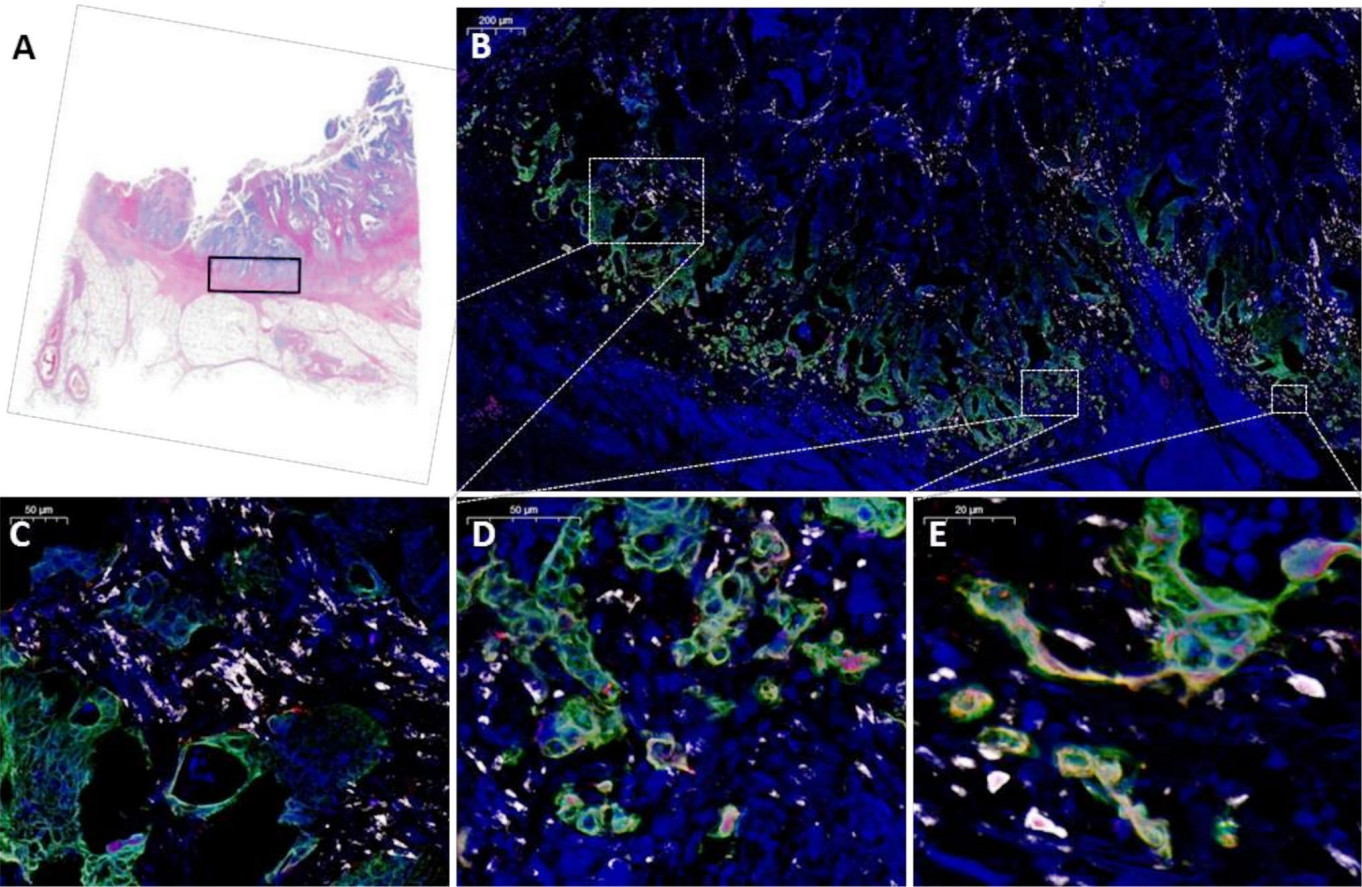
Image acquisition, identification of Region of Interest for confocal slide scanning. **a** The region of interest of approximately 25 mm^2^ was marked on a scanned H&E-stained slide. **b** After completion of the multiplex staining procedure, the slides were marked in the corresponding areas and images were obtained by confocal scanning microscopy. An example of the digital image is shown in **b** overview at × 5 objective of the merged image with cytokeratin (green), miR-21 (white), laminin-5γ2 (red) and DAPI (blue). **c**–**e** Demonstrations of different magnifications at × 20, × 40 and × 80, respectively

The areas of interest varied from 10 to 40 mm^2^ resulting in images of 15–55 GB each. The images were evaluated with CaseViewer software (3DHISTECH Ltd., Budapest, Hungary). During the image viewing processes, the variation in signal intensities between the three fluorophores (FITC with high intensity, Cy3 with low intensity and Cy5 with high intensity) caused overlay in Cy3 and Cy5 signals (bleed-through). Thus, the most intense stromal miR-21 ISH signal (Cy5) also emitted in the red filter and could be misinterpreted as laminin-5γ2 staining. Since our focus was on cytokeratin-positive adenocarcinoma cells (green fluorescence) in which the miR-21 signal was much weaker than in the stromal cells, red filter bleed-through was not considered a problem. An example of the image acquisition process is shown in Fig. 1.

### Evaluation of the multiplex fluorescence images

The digital images from 16 cases were evaluated. The miR-21 signal was assessed at the periphery of the invasive tumor front by combining the images for pan-cytokeratin and miR-21. The evaluation was performed manually on one or both scans from each case in extended focus mode. For the assessment of the tumor budding cells, the cytokeratin stained image (green channel) was used to identify the budding hot spots. This was performed at low magnification and three 40 × fields of views (area = 0.305 mm^2^) were drawn in high budding areas (Supplementary Fig. S1) using the integrated annotation software. The choice of three fields of view was considered appropriate, in that 1 high power field (HPF) may be used for biopsies and 10 HPFs for the surgical specimens of colorectal adenocarcinoma [19]. The adverse clinical association of high tumor budding has been established on scores in the most tumor budding dense areas [23], and such areas were therefore selected for miR-21 evaluation. The recently established guidelines recommend the assessment of tumor budding in one hotspot of 0.785 mm^2^ at the invasive front and the presence of ≥ 10 buds is considered high budding [25]. In this study, tumor budding was evaluated in an area slightly larger (0.915 mm^2^) than the recommended area.

The total number of tumor budding cells was counted according to the current definition (≤ 4 cells in a bud) [25] and is indicated in Table 1 (average = 73) along with the fraction of miR-21 positive cells. In each HPF, the number of tumor budding cells was annotated. Tumor budding cells located on the circular perimeter of the field were included if more than half of the cell was located within the perimeter. First, the total number of cytokeratin-positive tumor budding cells was counted. Secondly, focusing solely on the annotated cells, the presence or lack of miR-21 signal (white channel), laminin-5γ2 (red channel) and localization of miR-21 and laminin-5γ2 was evaluated. All information was noted in an Excel spread sheet. Annotations were indicated on the extended focus image, which is the virtual image composed of the confocal layers (the z-stack). The z-stack mode software tool was used to view the images in superior and inferior direction.

**Table 1:** Evaluation of miR-21 in cytokeratin-positive tumor budding cells

Case	Stage^a^	Dominant miR-21 tumor pattern	Focal miR-21 expressing carcinoma cells^b^	Total tumor budding cells^c^	miR-21 positive tumor budding cells (%)^d^	miR-21 and laminin-5γ2 co-localization (%)^e^	metachronous distant metastasis during 5 year follow-up
1	II	Stromal	No	59	0 (0)	0 (0)	No
2	II	Stromal	No	71	0 (0)	0 (0)	No
3	II	Stromal	No	138	0 (0)	0 (0)	No
4	II	Stromal	No	52	4 (7.7)	1 (1.9)	No
5	II	Stromal	No	72	6 (8.3)	1 (1.4)	Yes
6	III	Stromal	No	95	0 (0)	0 (0)	Yes
7	III	Stromal	No	155	0 (0)	0 (0)	No
8	III	Stromal	No	62	0 (0)	0 (0)	No
9	III	Stromal	No	80	1 (1.3)	0 (0)	Yes
10	III	Stromal	Yes	19	1 (5.3)	0 (0)	Yes
11	III	Stromal	Yes	81	8 (9.9)	6 (7.4)	No
12	III	Stromal	No	81	9 (11.1)	8 (9.9)	No
13	III	Stromal	Yes	82	11 (13.4)	5 (6.1)	No
14	III	Stromal	Yes	50	13 (26.0)	2 (4.0)	Yes
15	III	Epithelial^d^	–	37	10 (27.0)	3 (8.1)	Yes
16	III	Epithelial	–	52	22 (43.1)	3 (5.9)	Yes

^a^Stage II is characterized by absence of lymph node metastasis, stage III by the presence of lymph node metastasis

^b^Excluding tumor budding cells

^c^Counted in three high power fields

^d^Percentage of total number of tumor budding cells

^e^In FISH primarily epithelial miR-21 expression, while CISH showed global weak stromal expression with focal epithelial expression at the invasive front

### Statistical analysis

Fisher’s exact test was used to examine possible associations between miR-21 expression and clinico-pathologic characteristics. Statistical analyses were performed in STATA version 14.0 (StataCorp, College Station, TX, USA), and p values ≤ 0.05 were considered statistically significant.

## Results

### MiR-21 expression in colon cancer by CISH

**Fig. 2:**
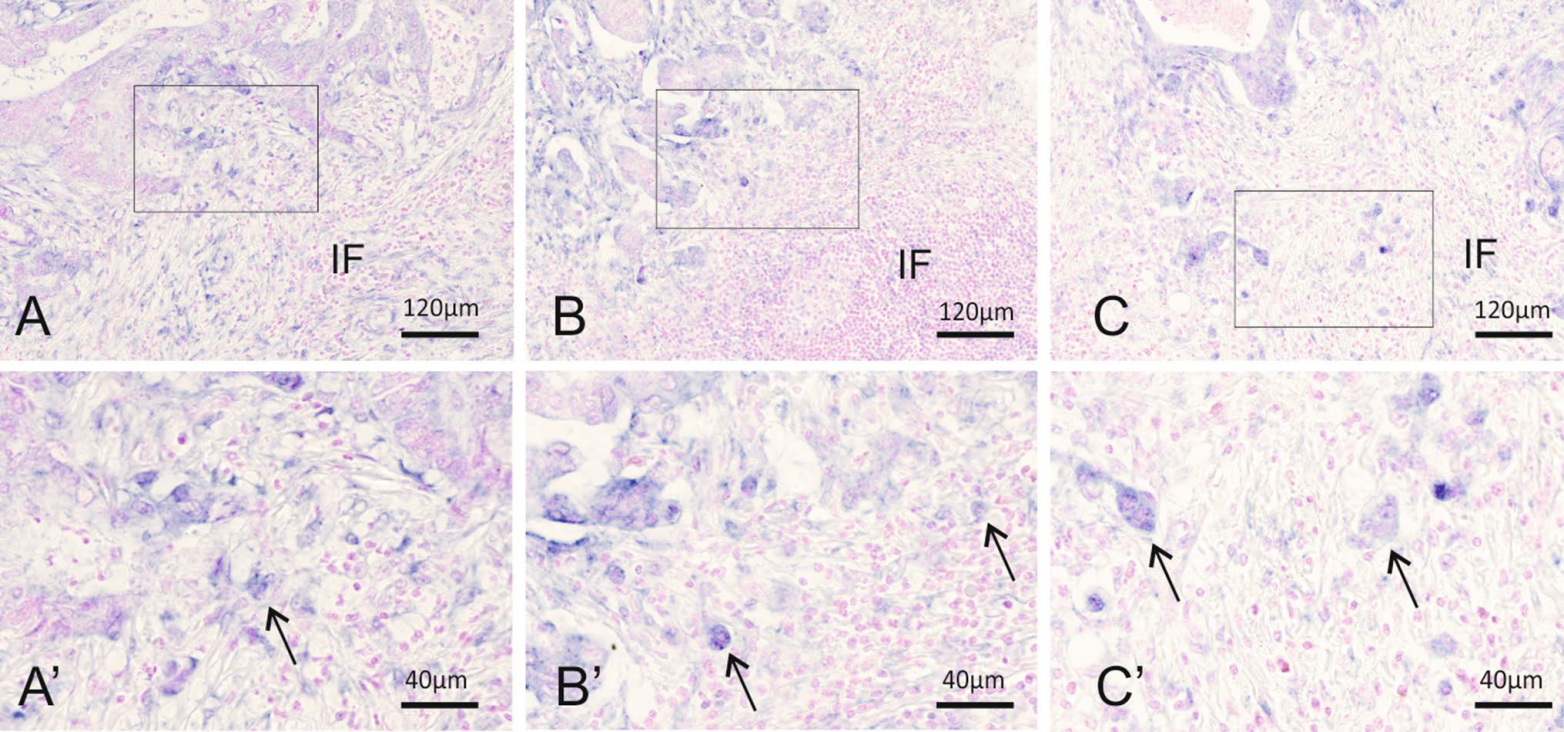
Examples of CISH miR-21 expression patterns in the tumor periphery. **a, a**′ Case with dominant stromal miR-21 expression where the tumor budding cells are difficult to distinguish from the miR-21 positive stromal cells. Close-up showing a possible tumor budding cell (arrow) among many miR-21-expression stromal cells. **b, b**′ Case with few miR-21 positive tumor budding cells (arrows) with some miR-21 expression in the surrounding stromal cells. **c, c**′ Case with dominant epithelial miR-21 expression. miR-21 expression is evident in the tumor budding cells (arrows). *IF* the invasive front of the colon adenocarcinoma

To characterize the expression of miR-21 in our sample cohort, 58 FFPE samples were stained by standard miR-21 CISH [8]. Fifty-six out of 58 colon adenocarcinomas were successfully stained, and the miR-21 CISH signal was present in all cases. Predominantly, the miR-21 signal was found in the cancer-associated fibroblast-like stromal cells, but was also seen in the adenocarcinoma cells both more widely in the cohesive adenocarcinoma compartment and locally within the tumor budding cells (Fig. 2).

The miR-21 CISH signal in the stromal cells varied locally both intra- and intertumoral. High stromal staining score was found in 47 cases (84%), and low stromal staining score in the remaining nine cases (16%). Thus, in all cases the miR-21 positive fibroblastic cells represented an internal positive control for the miR-21 CISH assay. A statistical association between male gender and high miR-21 stromal expressing cancers was found (*p* = 0.02), which is in agreement with our previous study [8]. In 34% of the adenocarcinomas, the miR-21 stromal expression declined from the tumor center to the periphery, while the opposite pattern was not observed. The declining stromal expression pattern was associated with stage III cancers and high budding (*p* values = 0.014) (Supplementary Fig. S2). We believe that the pattern may be related to miR-21 upregulation in smooth muscle that has been invaded by cancer cells and converted to myofibroblastic cells, which is in accordance with stage III cancers having traversed the muscularis externa and invaded deeper parts of the colon wall.

Focusing on the expression of miR-21 in the adenocarcinoma cells in general, high miR-21 expression was observed in ten tumors (18%). The expression was seen both in the tumor center and at the invasive front. A declining miR-21 expression in the adenocarcinoma cell from the center to the invasive front was seen in one case, while an upregulation from low to high expression was present in three other cases. The presence of miR-21-expressing adenocarcinoma cells was associated with left-sided cancers (*p* = 0.035). A difference between left and right sided cancers may not be surprising since those cancers are known to be clinically different both due to molecular and prognostic profiles [38].

We then sought to evaluate miR-21 expression in tumor budding cells. However, as we suspected, the stromal miR-21 expression prevented an unambiguous identification of the miR-21 expressing tumor budding cells. While a few miR-21 positive tumor budding cells could be identified as large single cells or small cell clusters with enlarged nuclei, these cells were often located among multiple miR-21 positive fibroblastic stromal cells in the micro-environment of the invasive tumor front making definite identification challenging. Thus, it was only possible to identify miR-21 positive tumor budding cells with certainty in the subset of cases with low miR-21 stromal expression (Fig. 2c–c′).

### MiR-21 expression in tumor budding cells assessed in confocal images

As unambiguous identification of miR-21 positive tumor budding cells in the CISH stains was not feasible, 20 cases were selected for characterization by multiplex immunofluorescence [34]. miR-21 ISH was combined with pancytokeratin immunohistochemistry for tumor budding cell identification and with laminin-5γ2, a well-established marker of tumor budding cells [39, 40]. An in vitro study has also suggested that laminin-5γ2 is positively regulated by miR-21 [41]. Digital images of the invasive front area were obtained by confocal slide scanning microscopy. The images covered 10–40 mm^2^ in a z-stack of 12 levels with a 0.4 µm distance. The assessment of the immunofluorescence stained slides was possible in 16 cases of which 12 displayed high tumor budding (Table S1).

**Fig. 3:**
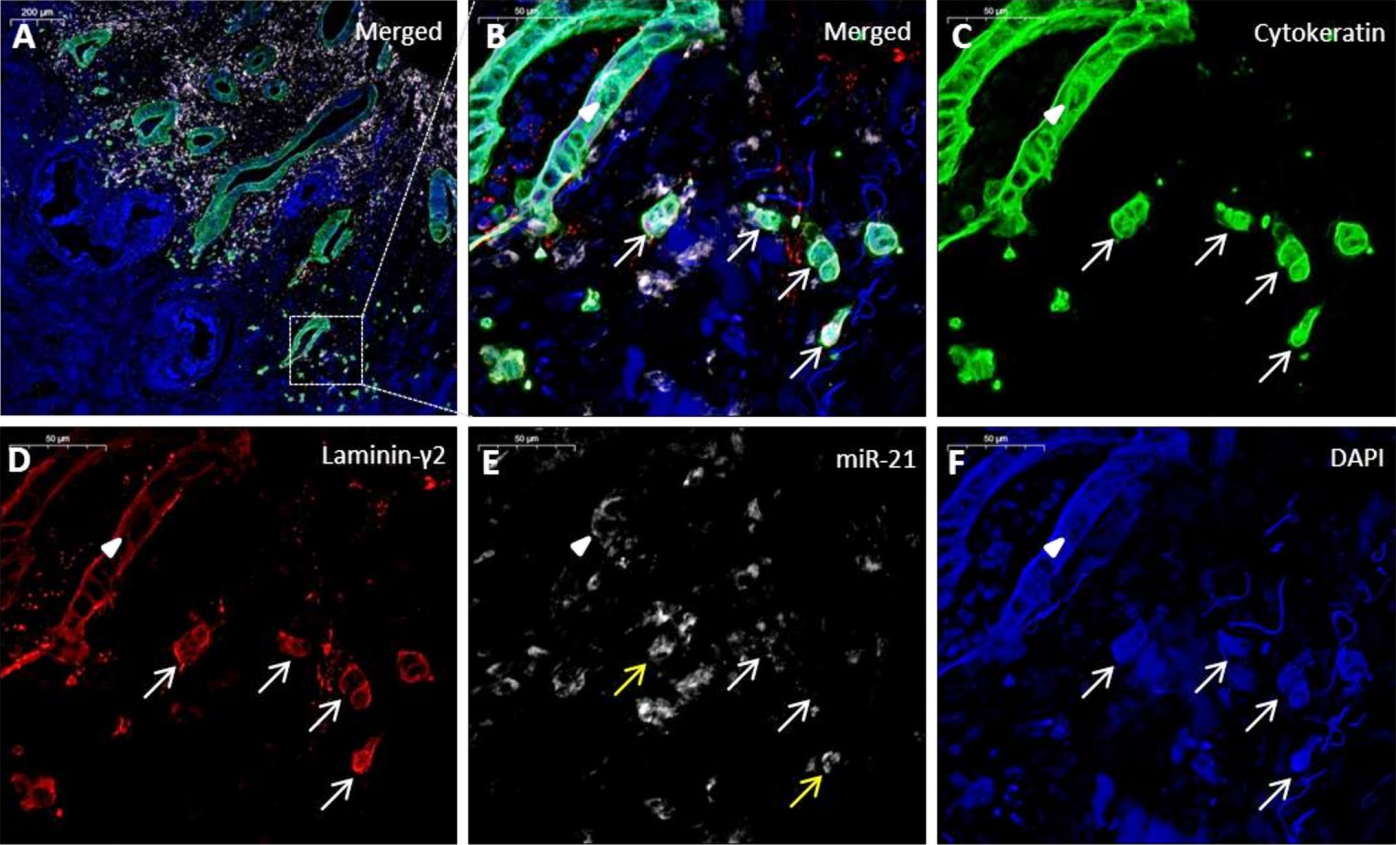
Colon cancer specimen with miR-21 positive tumor budding cells. **a** Merged image of the tumor periphery, cytokeratin (green), miR-21 (white), laminin-5γ2 (red) and DAPI (blue). **b** Magnification of an area containing tumor budding cells (arrows) and a malignant glandular structure (arrowhead). **c** Single channel image for cytokeratin. **d** Single channel image of laminin-5γ2. The adenocarcinoma cells show laminin-5γ2 overexpression. **e** MiR-21 expression is only seen in a few tumor buds (yellow arrows) and focally in the malignant gland structure (arrowhead). **f** DAPI image

In 14 cases, miR-21 expression was, as expected, found primarily in the fibroblast-like stromal cells of the cancers, whereas two cases showed predominantly expression in the carcinoma cells. This confirmed the observations in the CISH processed slides described above. MiR-21 FISH signal was present in all 16 cases as an inhomogeneous, diffuse cytoplasmic and nuclear signal (Fig. 3). The stromal miR-21 signal intensity was decreased at the invasive front, as also observed using CISH. We also noted that the miR-21 staining was reduced in the vicinity of the cytokeratin-positive tumor budding cells in the majority of the cases (data not shown).

The number of cytokeratin-positive tumor budding cells was counted in three HPFs (see Supplementary Fig. S1). The mean tumor budding cell count was 73.8 ± 8.6 (range 19–155), the number of miR-21 positive (and cytokeratin-positive) budding cells 5.3 ± 1.4 (range 0–18), and laminin-5γ2-positive (and cytokeratin-positive) cells 44.2 ± 11.2 (range 0–155) (Table 1). Co-localization of miR-21 and laminin-5γ2 was limited to a small fraction of tumor budding cells (Table 1). Ten cases out of 16 presented with miR-21 positive tumor budding cells (range 1.3–43.1%), and those with the highest fraction of positive tumor budding cells were stage III cancers (Table 1). This was not related to metachronous distant metastasis development.

The miR-21 staining pattern in the stromal cells and cohesive part of the tumors varied considerably: two cases had predominant miR-21 signal in the adenocarcinoma cells with only discrete expression in the stromal cells, while two other specimens exhibited focal expression in the cohesive part of the adenocarcinoma at the invasive front. One case showed exclusive miR-21 signal in the budding cells. Taken together, these observations indicate that miR-21 expression in adenocarcinoma cells is highly variable among tumors and that the expression in tumor budding cells is a recurring characteristic of this cell entity.

**Fig. 4:**
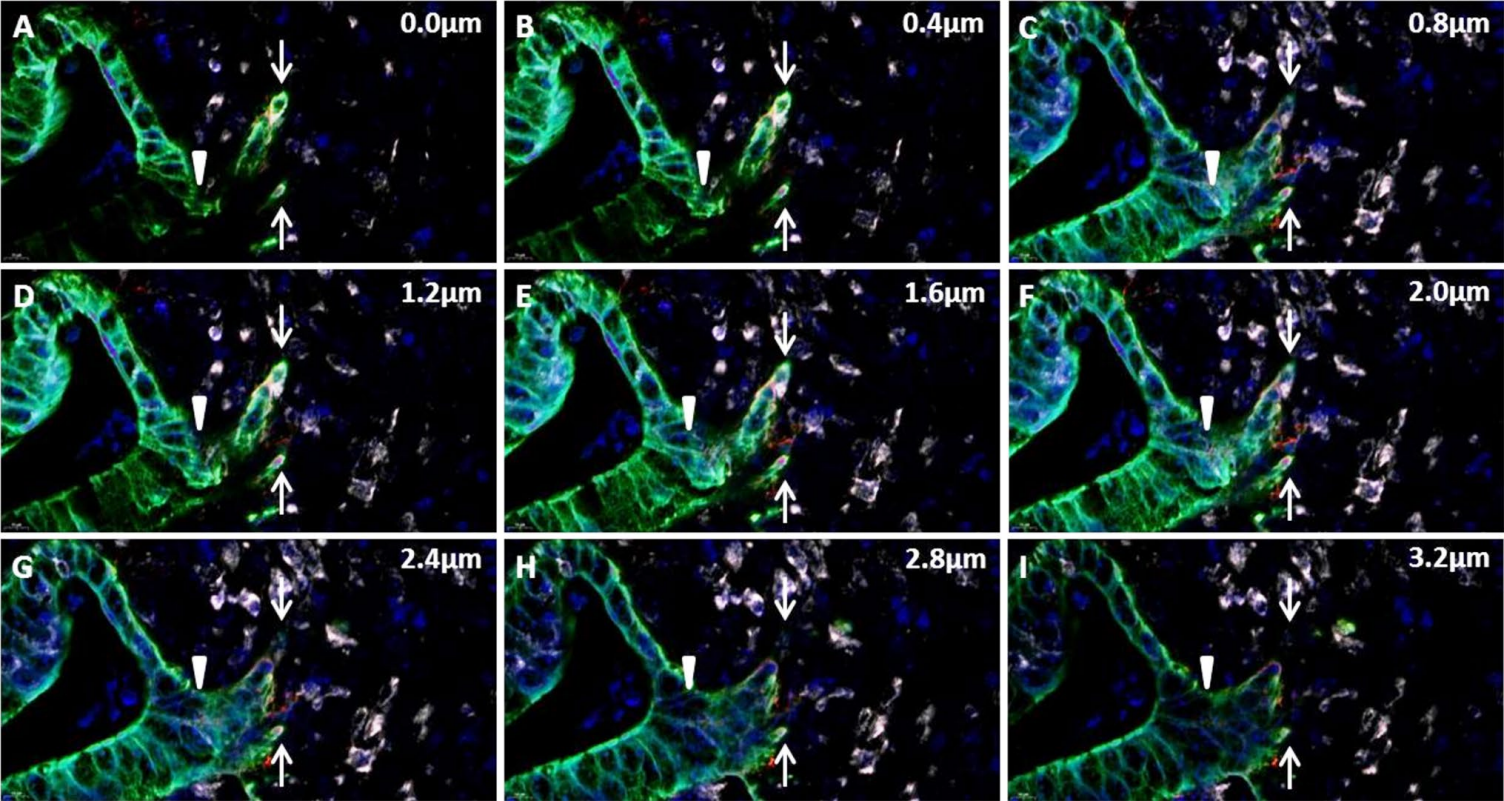
Tumor cell budding in confocal stack of images. Example of a confocal stack of images covering 3.2 µm in the z-axis in the tissue section, acquired from a digital whole slide of an adenocarcinoma tissue section stained for miR-21 (white), cytokeratin (green) and laminin-5γ2 (red). At baseline (0.0 µm) a budding cancer cell event (white arrow) and a malignant gland structure is noted. The stack reveals direct connection between the gland and budding cell event in the stack at 2.4–2.8 µm, identifying the tumor budding cells as a ‘branching event’

The use of the confocal image stack allowed the evaluation of not only miR-21 and cytokeratin-positive tumor budding cells but also the budding process itself. In these analyses, we noted that some islands of cytokeratin-positive tumor budding cells were in fact connected to the cohesive, malignant glandular structures (Fig. 4 and Fig. S2). It may be considered that, rather than separated islands of tumor cells, some of the tumor budding cells could actually represent ‘branches’ of tumor cells, connected to the cohesive tumor compartment.

## Discussion

While a number of methods are suitable for measuring microRNA expression in tissue samples, the microscopic visualization of microRNA is only possible by ISH [42]. Knowledge of the localization of microRNA expression in situ, and additional analysis of co-localization with cell markers or their target proteins, are essential for understanding their tissue-related roles and for characterizing their mechanisms of action. MiR-21 is among the most studied microRNAs [7]. Its consistent upregulation in cancer is well-established, and it is evident that various expression patterns, comprising fibroblastic cells and cancer cells, exist in different tumors, for example lung and breast cancer [43–46]. In colorectal cancer, we have observed that miR-21 expression in tumor budding cells appears to be an occasional phenomenon [8, 9]. The implication of miR-21 expression in this cell population is, however, of particular interest since tumor budding cells are known to be associated with increased risk of metastasis and poor survival [23, 47].

In this study, miR-21 expression was first evaluated in a sample set of 58 colon cancers by CISH. The expression patterns found in our sample set were in concordance with those reported in earlier miR-21 CISH studies of colorectal cancer [8–10]. Next, a subset of samples was selected for a more detailed evaluation of the miR-21 positive tumor budding cells. This was achieved by combining miR-21 fluorescence ISH with immunofluorescence staining [36] followed by examination of digital images obtained by confocal slide scanning microscopy. Using this approach allowed unambiguous identification of miR-21 positive cancer cells compared to the CISH stained slides suggesting that examination of the confocal digital slides can provide more profound observations than CISH.

The combination of multiplex fluorescence staining and confocal slide scanning microscopy offered several advantages. First, the cytokeratin-based identification of the budding cells ensured that miR-21 expression was evaluated in the appropriate cell population. This reduced the risk of misinterpreting stromal cells for tumor budding cells, as stromal cells are cytokeratin-negative. Second, the addition of another primary antibody, in this case against laminin-5γ2, allowed further characterization of the EMT process in relation to the miR-21-positive cells. Although, miR-21 was found not to be associated with laminin-5γ2 (discussed below), the additional antibody staining can be substituted by interesting alternatives such as E-cadherin and β-catenin both of which showing deregulated expression related to the invasive properties of tumor budding cells [14]. Third, confocal digital slides obtained by fluorescence scanning microscopy provided images with low background and autofluorescence signals in the FFPE sections. This was partly enabled by the combination of narrow band excitation (LED) light source, structured confocal imaging and narrow band fluorescence filters. Additionally, a water immersion lens with large numerical aperture allowed high resolution in the confocal layers, and image acquisition procedures with concomitant post-processing were applied for signal refinement. Fourth, digital slides containing four fluorescence stains (DAPI, FITC, Cy3 and Cy5) were easily evaluated and could be examined and shared repetitively. Digital zooming allowed examination at high magnification with the global position of the area in an image insert showing the whole slide. This ensured that orientation in large samples could be maintained, whereas in traditional epifluorescence microscopy orientation is easily lost when evaluating slides at 20x and 40x objective lenses. A fifth, noteworthy advantage was the optical, confocal stacks of images, which allowed a more accurate view of 3D structures [48]. Although the sections were only 5 µm in thickness, the ability to move inferiorly through the image proved valuable in cases with miR-21 signals from underlying stromal cells as well as in the identification of the budding cells. In this study, the evaluation of the whole z-stack unexpectedly revealed that a number of typical tumor budding cells were actually linked to the cohesive malignant glands. Thus, some tumor buds appeared to be an event of cancer cell branching, a phenomenon also reported by others [49]. This observation was not pursued any further in this study as the size of tumor budding cells (20–30 µm) would require thicker sections, optimally, to see both the tip and the connection to the cohesive tumor structure. It does, however, support the interesting assumption, that tumor budding cells could represent invasion by collective migration rather than single cell migration [49], but does not alter that tumor budding is indicative of an aggressive phenotype in a clinical setting [23].

The digital option proved advantageous, especially for inexperienced users of a fluorescence microscope, where familiarization can be time-consuming. Using the image viewer software, the slide evaluation was performed on a computer screen, which allowed for overview, enhanced zooming options, and easy annotations facilitating the evaluation process. However, the large image files are a limiting factor for older or smaller PCs. In our study, an area of approximately 10–40 mm^2^ resulted in file sizes ranging from 15 to 55 GB. These were easily handled by the v2.0 viewer software, but required hard drives with sufficient capacity.

We found that cases with the highest fraction of miR-21 positive budding cells were all stage III cancers defined by the presence of regional lymph node metastasis but no distant metastasis. It should be noted that the selected cases only included adenocarcinomas with high budding, and that the analysis was only performed on a subset of cases. Thus, our observation may not apply to stage II and III cancers with low tumor budding. No obvious relationship between miR-21 positive tumor budding cells in stage III cases and the development of subsequent distant metastasis was observed, but the study was performed on a small number of cases on which a possible association might not have revealed itself. Additional studies are needed to determine if the increased frequency of miR-21 positive tumor budding cells in stage III cancers does in fact reflect a more aggressive tumor phenotype, or if it is simply associated with the natural progression of the tumor. Our findings suggest that the more detailed information obtained from multiplex stained slides contributes to the understanding of the role of miR-21 in local cancer cell invasion.

Tumor budding cells differ both morphologically and molecularly from the cancer cells located in the central parts of the adenocarcinoma and are assumed to reflect cells in EMT [50]. These un-polarized single or small groups of carcinoma cells exhibit loss of E-cadherin and EpCam, but upregulated nuclear β-catenin and cytoplasmic laminin-5γ2 corresponding to the changes found in EMT [13]. Recently, we also found that the putative EMT-suppressor miR-200b is decreased in tumor budding cells [34]. Several in vitro studies suggest that also miR-21 plays a role in EMT [41, 51, 52]. Cottonham et al. showed that in LIM 1863 colon adenocarcinoma cells growing as organoids, TGF-β-induced EMT resulted in upregulation of miR-21 and increased mRNA levels of EMT-markers including laminin-5γ2 and matrix metalloproteinase-7 [41]. Such a correlation was not found between miR-21 and laminin-5γ2 in the present study. There may be several explanations for this discrepancy. We detected laminin-5γ2 at the protein level, while the effect in the study by Cottonham et al. was found at the mRNA level. In addition, their finding was made in cultured cells, whereas the dynamics in tumor budding cells in vivo is likely a much more dynamic process. Thus, it cannot be excluded that miR-21 may indirectly regulate laminin-5γ2 expression at specific stages of the EMT process, but it is also possible that miR-21 does not affect laminin-5γ2 expression in this cell population in vivo. It is, however, still likely that miR-21 may act through other well-known EMT-markers that were not examined in this study. In the metastatic breast cancer cell line MCF-7, re-expression of miR-21 increased cell motility and invasion with concomitant decreased E-cadherin and increased mesenchymal markers such as Vimentin and N-cadherin [51, 52]. Similarly, colon adenocarcinoma cell lines HTC116, CacoH2 and RKO, with high endogenous miR-21, showed low levels of E-cadherin [53]. Transfection of anti-miR-21 into high miR-21 expressing colon adenocarcinoma cells lines HCT116, RKO, SW-480 and LS174T cells, and a miR-21 mimic into low miR-21 expressing DLD-1 cells, increased apoptosis and reduced cell growth and invasiveness [53–55]. Kang et al. found that high stromal miR-21 and low E-cadherin was correlated [10]. Thus, one of the mechanism in which miR-21 may participate in the metastatic process could be via promotion of EMT in carcinoma cells. Interestingly, a recent study combining miR-21 ISH with cytokeratin-based immunocytochemistry showed that circulating tumor cells from 11 patients with different metastatic cancer types, including colon cancer, all co-expressed miR-21 and cytokeratin, while no cytokeratin-positive-miR21-negative circulating tumor cells were found [56]. Taken together, the observations suggest that the expression of miR-21 in tumor budding cells may protect the cells against apoptosis, that miR-21 is a marker of EMT and circulating tumor cell-precursors, and that increased miR-21 expression in tumor budding cells facilitates migration, intravasation and metastasis.

For future methodological improvements some aspects should be elaborated on in the multiplex staining procedure. For example, the cytokeratin fluorescence signal appeared stronger in the tumor periphery than in the central areas, contrary to what would be expected biologically. This may likely be caused by a combination of experimental and biological factors, including antigen accessibility, differential cytokeratin expression, pretreatment and signal amplification. Since the evaluation of the samples was intended to focus on the tumor periphery, the variation in the cytokeratin staining intensity was not considered an issue in this study. For studies relying on a comparison between the tumor center and periphery, an alternative cytokeratin antibody may prove more useful. In addition, selecting alternative fluorophores that have minimum overlap in the emission specters may eliminate potential emission bleed-through, as observed for the intense Cy5 signal from miR-21 that emitted in the Cy3 filter for laminin-5γ2. In this case, it was a minor problem because (cytokeratin-positive) tumor budding cells always showed a relatively low miR-21 staining intensity. Further studies could also benefit from an evaluation of intra- and inter-observer variation which was not performed in the present study due to its descriptive and hypothesis generating nature. Overall, the combination of multiple fluorescence staining and confocal scanning microscopy was found to be unique for resolving and evaluating the complex staining patterns.

In conclusion, we have developed a multiplex staining method in order to better characterize the miR-21 expressing tumor budding cells in stage II and III colon adenocarcinomas. By examination of high resolution 3D digital images obtained by confocal scanning microscopy, we found co-expression with cytokeratin, but rarely with laminin-5γ2, and an increased frequency of miR-21 positive tumor budding cells in stage III compared to stage II cancers. These findings may reflect aspects of tumor progression or the presence of a particular pro-metastatic cell population. Whether the biological function of miR-21 in the tumor budding cells supports a particular hostile cellular phenotype is yet to be elucidated. For therapeutic targeting of miR-21 expressing colorectal cancer cells, it is of particular interest that anti-sense therapy targeting tumor cells expressing miR-21 has been reported to reduce in vivo growth of e.g. myeloma and glioblastoma in experimental settings [57, 58].

## Electronic supplementary material

Below is the link to the electronic supplementary material.

Supplementary material 1 (DOCX 16 KB)

Supplementary Fig. S1 Tumor budding assessment in a multiplex stained slide. A) The multiplex images were evaluated at low magnification on the merged cytokeratin (green) and DAPI image (blue) to identify tumor budding hot spots. Three 40x objective fields of view (area= 0.305 mm^2)^) were drawn using the integrated annotation system. B) The cytokeratin-positive tumor buds were annotated and counted. Cells on the circular perimeter of the field of view were counted if more than half of the cell was found within the border. C) In the merged image, cytokeratin (green), miR-21 (white) and DAPI (blue), miR-21 expression was evaluated in the annotated cells only. In this particular case, no miR-21 signal was found in the cytokeratin-positive tumor buds. D) Represents the same image as in C, but including laminin-5γ2(red). The cytokeratin-positive cells were evaluated for laminin-5γ2 using a merged image supplemented with the red channel only (not shown), and the total number of laminin-5γ2 cells and cells with miR-21 and laminin-5γ2 co-localization were recorded (TIF 9039 KB)

Supplementary Fig. S2 A) Stage III colon adenocarcinoma showing decreased expression of miR-21 from the tumor center towards the invasive front. B) Strong stromal miR-21 expression in a stage II colon adenocarcinoma (TIF 6940 KB)

Supplementary Fig. S3 Example of tumor cell budding confocal stack of images. Another example (with reference to Fig. 4) of tumor cell branching, tentatively interpreted as tumor budding, identified in a confocal stack of images covering 3.2 µm in the z-axis of the tissue section, acquired from a digital whole slide of a colon adenocarcinoma tissue section, stained for miR-21 (white), cytokeratin (green) and laminin-5γ2 (red) (TIF 2809 KB)
