# Supplementary material for: miR-21 expression analysis in budding colon cancer cells by confocal slide scanning microscopy
Source: Clin Exp Metastasis. 2018 Oct 25;35(8):819–30. doi: 10.1007/s10585-018-9945-3 (PMC6267652; doi:10.1007/s10585-018-9945-3)
Supplement: Supplementary file 1 — Supplementary material 1 (DOCX 16 KB) [file 10585_2018_9945_MOESM1_ESM.docx]

**Table S1** Clinico-pathological data for the studied group and subgroups

| **Variable** | | **Tumors stained with CISH n=58 (%)** | **Tumors stained with multiplex fluorescence**  **n=16 (%)** |
| --- | --- | --- | --- |
| **Gender** |  |  |  |
|  | Female | 39 (67) | 14 (87.5) |
|  | Male | 19 (33) | 2 (12.5) |
| **Age (years)** |  |  |  |
|  | Mean | 73 | 75.6 |
|  | Range | 49-98 | 49-90 |
| **Localization** |  |  |  |
|  | Right colon | 33 (57) | 8 (50) |
|  | Left colon | 25 (43) | 8 (50) |
| **Stage^*^** |  |  |  |
|  | Stage II | 36 (62) | 5 (31) |
|  | Stage III | 22 (38) | 11 (69) |
| **Mismatch repair protein** |  |  |  |
|  | Proficient | 48 (83) | 12 (75) |
|  | Deficient | 10 (17) | 4 (25) |
| **Tumor differentiation** |  |  |  |
|  | Low | 4 (7) | 5 (31) |
|  | Moderate | 42 (72) | 9 (62.5) |
|  | High | 12 (21) | 2 (12.5) |
| **Vascular invasion** |  |  |  |
|  | No | 50 (86) | 13 (81) |
|  | Yes | 8 (14) | 3 (19) |
| **Perineural invasion** |  |  |  |
|  | No | 48 (83) | 12 (75) |
|  | Yes | 10 (17) | 4 (25) |
| **Tumor budding^**^** |  |  |  |
|  | Low | 38 (35) | 4 (25) |
|  | High | 20 (64) | 12 (75) |
| **Recurrence** |  |  |  |
|  | No | 43 (74) | 9 (56) |
|  | Yes | 15 (26) | 7 (44) |

^*^ Stage II is characterized by absence of lymph node metastasis, stage III with presence of lymph node metastasis but no distant metastasis

^**^Low and high budding was defined as <100 or ≥100 tumor buds (≤4 cells) in a total of 10 high power fields
