# Supplementary figures and images for: miR-21 expression analysis in budding colon cancer cells by confocal slide scanning microscopy
Source: Clin Exp Metastasis. 2018 Oct 25;35(8):819–30. doi: 10.1007/s10585-018-9945-3 (PMC6267652; doi:10.1007/s10585-018-9945-3)

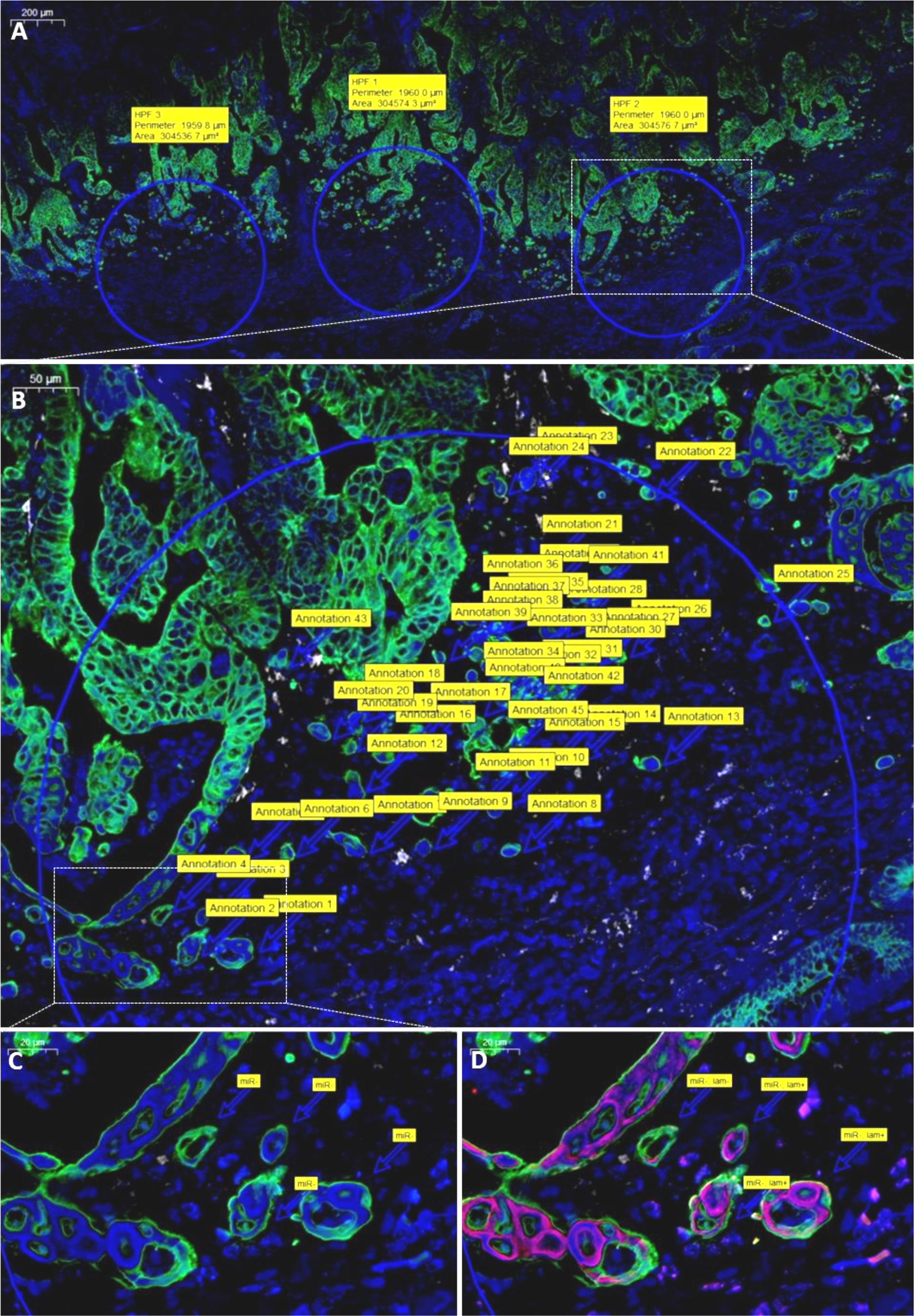

Supplement: Supplementary file 2 — Supplementary Fig. S1 Tumor budding assessment in a multiplex stained slide. A) The multiplex images were evaluated at low magnification on the merged cytokeratin (green) and DAPI image (blue) to identify tumor budding hot spots. Three 40x objective fields of view (area= 0.305 mm2)) were drawn using the integrated annotation system. B) The cytokeratin-positive tumor buds were annotated and counted. Cells on the circular perimeter of the field of view were counted if more than half of the cell was found within the border. C) In the merged image, cytokeratin (green), miR-21 (white) and DAPI (blue), miR-21 expression was evaluated in the annotated cells only. In this particular case, no miR-21 signal was found in the cytokeratin-positive tumor buds. D) Represents the same image as in C, but including laminin-5γ2(red). The cytokeratin-positive cells were evaluated for laminin-5γ2 using a merged image supplemented with the red channel only (not shown), and the total number of laminin-5γ2 cells and cells with miR-21 and laminin-5γ2 co-localization were recorded (TIF 9039 KB) [file 10585_2018_9945_MOESM2_ESM.tif]

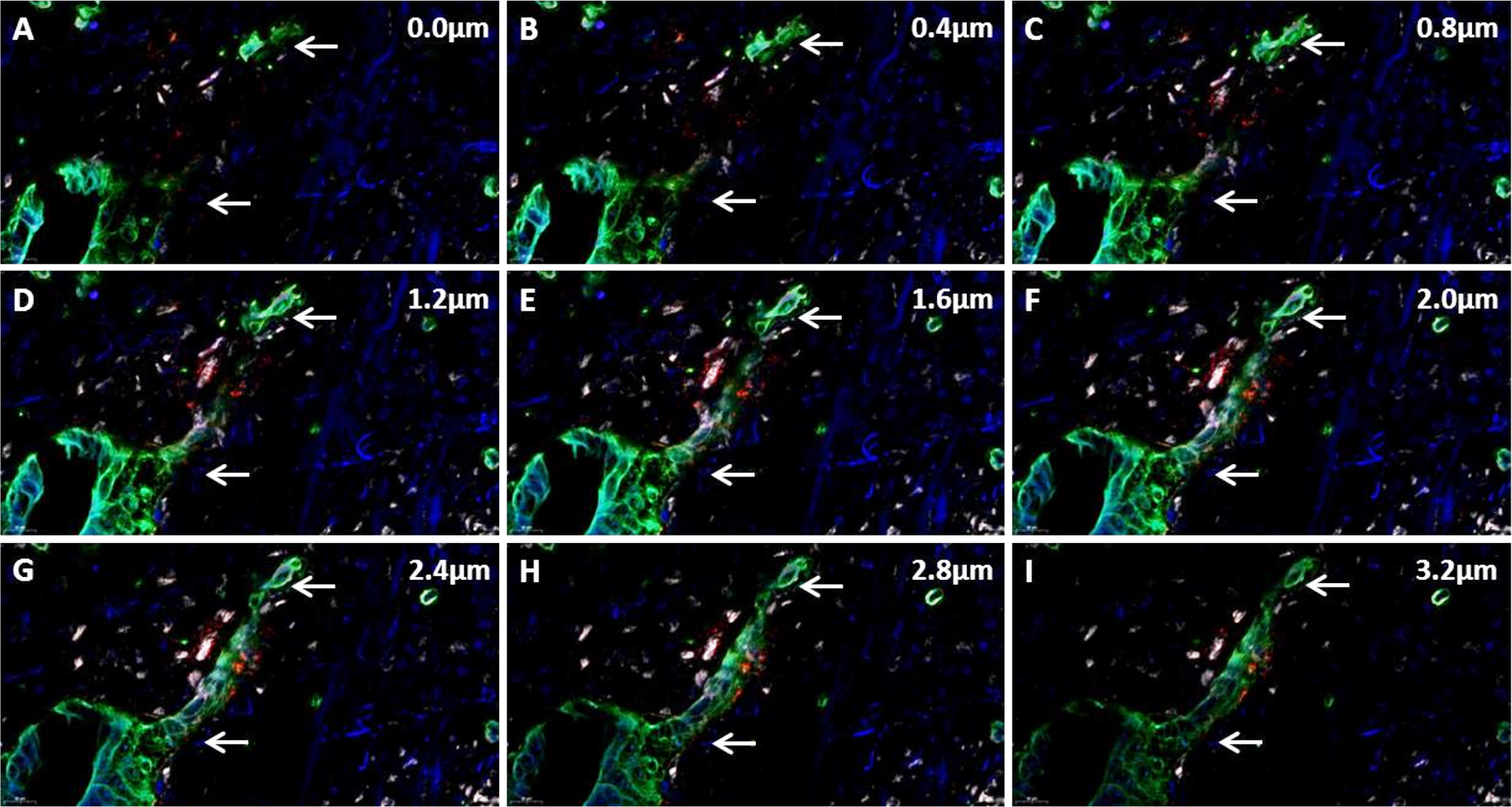

Supplement: Supplementary file 4 — Supplementary Fig. S3 Example of tumor cell budding confocal stack of images. Another example (with reference to Fig. 4) of tumor cell branching, tentatively interpreted as tumor budding, identified in a confocal stack of images covering 3.2 µm in the z-axis of the tissue section, acquired from a digital whole slide of a colon adenocarcinoma tissue section, stained for miR-21 (white), cytokeratin (green) and laminin-5γ2 (red) (TIF 2809 KB) [file 10585_2018_9945_MOESM4_ESM.tif]
